# Supplementary material for: Complexome profiling of the Chlamydomonas psb28 mutant reveals TEF5 as an early PSII assembly factor
Source: Plant Cell. 2025 Mar 17;37(6):koaf055. doi: 10.1093/plcell/koaf055 (PMC12164590; doi:10.1093/plcell/koaf055)
Supplement: koaf055_Supplementary_Data [file koaf055_supplementary_data.zip › psb28_interactive.zip!/plots/intensity/qualityPlots/peptides/TEH6_Cre16.g683350.t1.2.html]

### Cre16.g683350.t1.2

Trivial name: TEH6  
SeqCoverage [median]: 13.40  
Mass: 58.41 kDa  
  
 

| Sequence | MissedCleavage | Mass [Da] | wtIntensity | muIntensity | Score |
| --- | --- | --- | --- | --- | --- |
| FVLVAR | 0 | 703.44 | 0.00 | 377.88 | 78.00 |
| LEEPLELK | 0 | 969.54 | 461.10 | 0.00 | 65.04 |
| LTGQVVYVEGPVVR | 0 | 1514.85 | 504.34 | 0.00 | 93.26 |
| NVAPFSYEVGEHR | 0 | 1503.71 | 561.52 | 0.00 | 43.99 |
| TAAADVPVLLPQTPR | 0 | 1547.87 | 379.45 | 1271.31 | 76.65 |
| VASLAEVK | 0 | 815.48 | 0.00 | 494.31 | 58.92 |
|  |  |  | 1906.4 | 2143.5 |  |
